# The impact of COVID-19 on persons with concurrent mental health and substance use disorders: Marja Leonhardt

**DOI:** 10.1093/eurpub/ckac131.045

**Published:** 2022-10-25

**Authors:** M Leonhardt, L Lien

**Affiliations:** 1 Sykehuset Innlandet HF, Norwegian National Advisory Unit on Concurrent Substance Abuse and Mental Health Disorders, Brumunddal, Norway; 2 Department of Health and Social Science, Inland Norway University of Applied Sciences, Elverum, Norway; 3 Faculty of Health Studies, VID Specialized University, Oslo, Norway

## Abstract

**Background:**

The COVID-19 pandemic with its restrictions touched the daily life of most people. While everyday social life becomes difficult for citizens with economic and cultural capital, it becomes even worse for persons with mental health (MHD) and substance use disorders (SUD), who are particularly vulnerable to social exclusion. In this project, we aim to explore the impact of the pandemic on persons MHD/SUD, nearer, how the lockdown effected their daily life and further, the utilization of health care services under the consecutive waves of the pandemic.

**Methods:**

The project has two parts: First we conducted 17 individual interviews and one focus group with persons with MHD/SUD, using thematic analysis. Second, we merged the Norwegian Patient Register, the Register for Infectious Diseases and data from Statistics Norway. We matched data of 41500 individuals with MHD/SUD after gender, age and health region with a sample from the general population as a control group and study the health care service utilization under the consecutive waves of the pandemic in Norway in 2020-2021.

**Results:**

Within the qualitative study, we identified four interrelated main themes: (1) The COVID-19 outbreak as a perceived challenge, (2) A decline in mental health and well-being, (3) Increased substance use challenges, and (4) Diverse experiences with health and social services. The results show further that people with MHD/SUD have challenges with digital tools and/or do not have the appropriate equipment. Persons with MHD/SUD face greater barriers in accessing the health care system compared to the general population as a control group. Results of the register study are still preliminary.

**Conclusions:**

Persons with MHD/SUD face major challenges during the COVID-19 pandemic There is reason to believe that new pandemics will emerge in the future. In this context, it is essential to gain knowledge of how to care for vulnerable groups in society and how to reach them in emergencies.

**Key messages:**

• Continuous maintenance of low-threshold services for persons with MHD/SUD during a pandemic is essential.

• Improvement of digital skills of service users or alternatives to digital consultations should be considered.

